# Breech birth care: Number 1 – 2024

**DOI:** 10.61622/rbgo/2024FPS01

**Published:** 2024-03-15

**Authors:** Álvaro Luiz Lage Alves, Alexandre Massao Nozaki, Carla Betina Andreucci Polido, Lucas Barbosa da Silva, Roxana Knobel

**Affiliations:** Universidade Federal de Minas Gerais Hospital das Clínicas Belo Horizonte MG Brazil Hospital das Clínicas, Universidade Federal de Minas Gerais, Belo Horizonte, MG, Brazil.; Hospital do Servidor Público Municipal São Paulo SP Brazil Hospital do Servidor Público Municipal, São Paulo, SP, Brazil.; Universidade Federal de São Carlos Faculdade de Medicina São Carlos SP Brazil Faculdade de Medicina, Universidade Federal de São Carlos, São Carlos, SP, Brazil.; Hospital das Clínicas São Sebastião SP Brazil Hospital das Clínicas, São Sebastião, SP, Brazil.; Universidade Federal de Santa Catarina Faculdade de Medicina Florianópolis SC Brazil Faculdade de Medicina, Universidade Federal de Santa Catarina, Florianópolis, SC, Brazil.

## Key points

- It is essential to resume teaching external cephalic version and obstetric maneuvers in vaginal breech birth, both in lithotomy and in the vertical position.
- The adoption of strict protocols for planned vaginal breech birth correlates with a success rate of approximately 70% and adverse outcome rates of less than 7%. Fetal and neonatal morbidity and mortality are similar to those of a planned cesarean section.
- Pregnant women eligible for vaginal breech birth must agree to the mode of delivery, be at low risk of complications and have assistance of professionals with experience in vaginal birth of abnormal presentations and in obstetric maneuvers.
- Previous cesarean section and prematurity between 32 and 36 weeks are not absolute contraindications to vaginal breech birth and must be individually evaluated when deciding on the route of delivery.
- Neonatologists must be present at the birth of breech fetuses, and a complete neonatal examination must be performed.
- Posterior rotation of the fetal back, prolapse of the umbilical cord, deflection of the arms and/or cephalic pole and retention of the after-coming head are the main dystocias related to vaginal breech birth assistance. Every professional assisting vaginal breech births must be trained to adequately resolve these events.
- In vaginal breech birth in lithotomy, the main maneuvers to help in the delivery of the fetal pelvis are bidigital inferior traction on the inguinal region and the Pinard maneuver; for the release of the fetal trunk, those of Rojas, Deventer-Miler and Pajot; and for the release of the after-coming head, those of Mauriceau, Bracht, Champetier de Ribes and Prague and operative vaginal delivery with Piper's forceps.
- Non-lithotomic positions in vaginal breech birth are associated with reduced dilation and expulsion periods, need for maneuvers for fetal extraction, as well as reduced rates of cesarean sections and neonatal injuries.
- In vaginal breech birth assisted in the all fours position, the aspects to be observed during delivery of the fetal body include the fetal muscle tone of lower limbs, the correct rotation of the fetal trunk (fetal abdomen facing the maternal back), vascular engorgement of the umbilical cord, the presence of elbows and folds of the fetal chest and maternal anal dilatation.
- In vaginal breech births assisted in the all fours position, more than half of fetuses are delivered without the need for any maneuver. Usually, only two maneuvers may be necessary: one to help release the shoulders (180°-90° rotation) and another to release the fetal head (Frank's nudge).

## Recommendatios

- In order to prevent cesarean sections and dystocia associated with vaginal breech birth, the external cephalic version, when available, should be offered to pregnant women who reach term with their fetuses in breech presentation.
- The mode of delivery in breech presentation must be an informed choice, defined according to the values and preferences of the parturient woman, the experience, values and preferences of the care team, adequacy of obstetric and neonatal care conditions, and assessment of benefits and risks of approaches.
- Pregnant women without pelvic abnormalities or defects, fetuses in incomplete breech (Frank breech) or complete breech presentation, weighing between 2,000 and 4,000 g, excluding intrauterine growth restriction, anomalies susceptible to dystocia and hyperextension of the cephalic pole, in spontaneous labor or under planned induction at term, with maternal desire and consent, and an experienced obstetrician available are eligible for vaginal breech birth.
- A clinical obstetric examination with or without ultrasound must confirm the mode of breech presentation and fetal attitude upon admission of the parturient woman for birth care. Electronic fetal monitoring must be continuous. Amniotomy should not be performed, and a vaginal examination should always be performed after spontaneous amniorrhexis. Routine episiotomy is not indicated.
- Planned induction of breech birth is viable and has a high success rate, as long as it is performed between 37 and 38 weeks by experienced professionals, in places with resources to safely perform an emergency cesarean section and adequate neonatal care.
- In assisting vaginal breech births, neuraxial analgesia with minimal motor block or a pudendal nerve block can be used for pain relief. Oxytocin should be used in the prolonged latent phase and in transient post-anesthetic hypocontractility. Its use in prolonging the active phase must be individualized.
- Planned cesarean section for breech presentation is associated with increased short-term maternal morbidity and various risks in subsequent pregnancies. Long-term maternal and infant outcomes are similar to those with the vaginal route of birth. If the abdominal route is chosen, it should be planned for between 39+0 and 41+0 weeks, allowing for ideal physiological maturity of the fetus and the possibility of spontaneous version. Fetal position must be confirmed immediately before surgery.
- Cesarean section of breech fetuses must have a wide abdominal incision and hysterotomy. For extraction of term and borderline fetuses, low transverse segmental hysterotomy is indicated. For the extraction of premature breech fetuses, especially in extreme prematurity, longitudinal segmental or corporal hysterotomy is recommended.
- Breech fetuses with anomalies must have an individual diagnosis and definition of the route of delivery. Fetuses with macrocephaly (or other conditions leading to dystocia) considered viable must be extracted by cesarean section. Those with these conditions, although unviable, should preferably be selected for the vaginal route, which may require cephalocentesis.
- In breech births progressing to the vaginal route in the absence of qualified staff and/or adequate facilities, it is recommended to avoid voluntary or directed pushing, institute pharmacological uterine relaxation and provide transfer of care. If transfer is impossible, or when labor is imminent and/or in expulsion of the fetal buttocks or lower limbs, it is recommended to place the woman in labor on all fours position (Gaskin's position) or in a squatting position and do not apply traction on the fetus.

## Background

Breech presentation occurs in approximately 3% of full-term pregnancies. In breech birth, the vaginal route poses risks related to the slower delivery of the fetal body and the fact that the biparietal diameter is the last to be released, exposing newborns to greater morbidity and mortality. Various delivery maneuvers may be necessary to assist in the delivery of fetal diameters (bitrochanteric, biacromial and biparietal), especially in the lithotomy position, requiring specific knowledge and skills.^(1)^

Prevention of breech birth can be achieved through the external cephalic version (ECV), a procedure that can increase the frequency of parturient women admitted to labor with fetuses in cephalic presentation.^(1)^

Vaginal breech birth has been progressively being replaced by cesarean sections. We can say that biased conclusions from studies from the beginning of this century contributed to this scenario, since the recommendation of elective cesarean section as the safest practice for all fetuses in breech presentation was quickly and widely disseminated. Since then, elective cesarean sections, but also emergency cesarean sections performed in the active phase of labor, have become common in breech birth care. Consequently, vaginal breech births have progressively become the exception, often being conducted as obstetric emergencies.^(2)^

At the same time, practices to support vaginal breech birth, particularly in upright positions, have been studied and published, offering the possibility of rescuing this mode of delivery based on possibly more favorable results. Therefore, guidelines need to be corrected and updated, expanding the appropriate selection of women who can be assisted vaginally. It is also essential to resume teaching ECV and delivery maneuvers, both those already known and useful in lithotomy assistance and those more recently described for assistance in the vertical position.^(3,4)^

## What are the main types of breech presentation and which are most favorable to vaginal birth?

**Figure 1:**
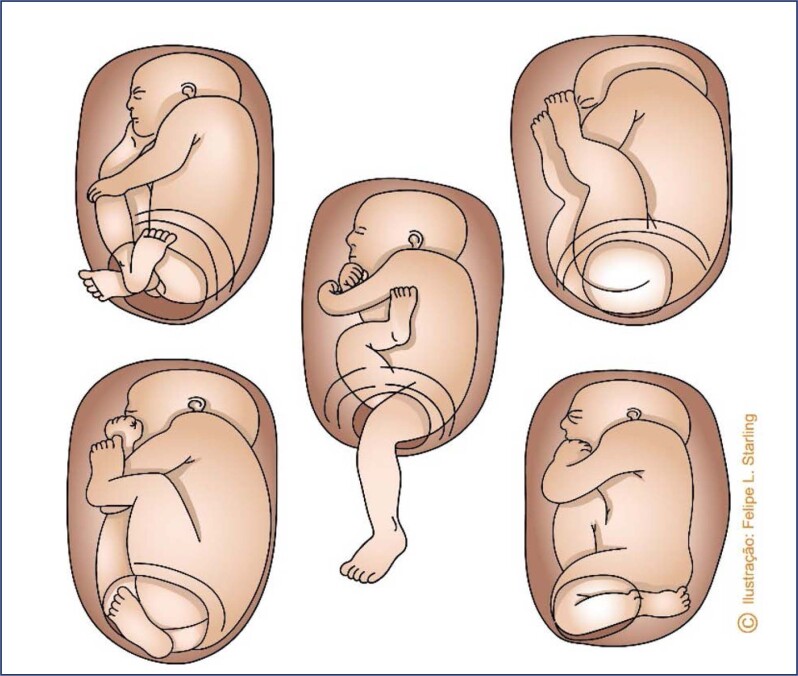
Types of breech presentation

Source: Illustration by Felipe Lage Starling (authorized).

Breech presentations can be incomplete or complete. Incomplete presentations are subdivided into the modes of frank breech, knees or footling. In frank breech, the hips are flexed and the legs are extended in front of the torso. In other incomplete breech presentations, parts of one or both lower limbs (knees and feet) are positioned inferiorly in relation to the hips. Complete breech presentations present flexed hips and knees, with feet crossed close to the buttocks (Figure 1). The most common breech presentation is the incomplete frank breech, with a frequency between 50% and 70%. Complete breech presentation has a frequency between 5% and 10%, and other incomplete presentations account for 10-40%.^(5)^

Frank breech and complete breech presentations are the most favorable to vaginal birth. In these presentations, the lowest plane of the fetal body contains the largest bitrochanteric diameter, which provides effective dilation of the soft parts of the birth canal and favors the descent and subsequent delivery of the biacromial and biparietal diameters. Therefore, the fetal thighs and trunk pass simultaneously through the birth canal, facilitating the release of shoulders and the head. The short-term morbidity of these presentations appears to be similar, although assistance with maneuvers are more necessary in complete breech presentation.^(5)^ Knee and footling mode incomplete breech presentations have the disadvantage of premature release of the lower limbs in relation to the fetal pelvis. Therefore, the limbs are easily released by an incompletely dilated cervix, or even by a pelvis of inadequate dimensions. This peculiarity often imposes difficulties for the immediately subsequent delivery of the bitrochanteric, biacromial and biparietal diameters, since they are significantly larger than the diameters of the lower limbs. Additionally, the early release of the lower limbs offers more space for umbilical cord prolapse, which can worsen a situation of hypoxia already imposed by the hindered release of the fetal trunk and head. Therefore, single fetuses with these presentations are not eligible for vaginal delivery, and this method of delivery is only acceptable if it is a second twin fetus, for which the possibility of successful foot extraction is greater.^(5)^

## What are the prevalence, pathogenesis, risk factors and risk of recurrence in breech presentation?

Due to the high mobility of the fetus in a uterine cavity containing a relative increased volume of amniotic fluid, breech presentation is a common occurrence in early pregnancy. The prevalence rates before 28 weeks and at term are, respectively, 20-25.7% and 3-4%.^(6)^ Fetuses in breech presentation can occur in any pregnancy, although in more than 15% of cases, they may be related to placental, fetal or maternal abnormalities. Fetuses with normal anatomy, activity, volume of amniotic fluid and placental location are positioned in cephalic presentation close to term, as this fetal position becomes more suitable for the uterine space (content/continent relationship; Pajot's law). When abnormalities are present in these variables, the probability of breech presentation increases.^(6)^ Multiple factors are related to the increase in breech presentation, including: preterm pregnancy, family history, uterine abnormalities (bicornuate and septate uteri, fibroids), placental location (previa and cornual placentas), multiparity (loss of abdominal wall tone, more rounded intrauterine space), oligohydramnios, polyhydramnios, contracted maternal pelvis, fetal anomalies (anencephaly, hydrocephalus, sacrococcygeal teratoma, cervical tumors), extension of fetal lower limbs, twin birth, fetal neurological diseases, maternal hypothyroidism, short umbilical cord, intrauterine fetal growth restriction, fetal asphyxia, primiparity, female sex, maternal anticonvulsant therapy and advanced maternal age.^(7)^ The risk of recurrence increases in percentages of 9%, 25% and almost 40% after, respectively, one, two or three consecutive breech pregnancies.^(8)^

## How should breech pregnancies be managed?

There is a consensus on the greater risk of asphyxia and trauma among breech fetuses born vaginally. For the minimization of these risks, the choice of mode of delivery must be defined according to the values and preferences of the parturient, the experience, values and preferences of the obstetrician, adequacy of obstetric and neonatal care conditions and assessment of benefits and risks of the approaches.^(1)^ Strategies for approaching the delivery of breech fetuses include:

1. ECV at the end of pregnancy before labor with an attempt at vaginal birth if the procedure is effective. If unsuccessful, offer a cesarean section;
2. ECV at the end of pregnancy before labor with an attempt at vaginal birth if the procedure is effective. In the event of failure, offer a vaginal birth attempt to pregnant women identified as low risk and with favorable criteria for this method of delivery. A cesarean section should be offered to patients who refuse an attempt at vaginal birth and to those identified as being at high risk for this method of delivery. Preferably, it should be performed after the start of labor or at 40 weeks;
3. Cesarean section without an attempt at ECV;
4. Attempt at vaginal birth without ECV for pregnant women identified as low risk and with favorable criteria for this method of delivery.^(1)^

Spontaneous delivery of breech fetuses can occur at any time, even after 40 weeks. Approximately 25% of breech fetuses at 36 weeks of pregnancy develop a spontaneous version until birth. Factors that reduce the likelihood of spontaneous version (and successful ECV) include fetal leg extension, oligohydramnios, short umbilical cord, fetal and/or uterine abnormalities, and nulliparity.^(9)^ With the aim to prevent cesarean sections and dystocia associated with vaginal breech birth, ECV should be offered to pregnant women who reach term with their fetuses persisting in breech presentation. The success of ECV is highly variable, with a pooled rate of 58%; ECV is not without risks, and the cesarean section rate after the successful procedure is higher than that for fetuses in spontaneous cephalic presentation. Complications are infrequent (rate of 6.1%), with emphasis on placental abruption, emergency cesarean section and stillbirth.^(10)^ Therefore, some women may choose cesarean section without attempting ECV. Pregnant women with a low probability of success in the version or an increased risk of fetal harm associated with the procedure may reasonably avoid attempting ECV and opt for a cesarean section.^(11)^ Another subgroup of patients may opt for attempted labor and vaginal breech birth without an ECV attempt. Therefore, pregnant women with a low probability of success in the version or at high risk of fetal harm associated with the procedure may also reasonably avoid ECV and choose to attempt a vaginal breech birth. This conduct must be in line with the general consensus that these pregnant women are at low risk for associated complications and assisted by an obstetrician with experience in vaginal breech birth.^(12)^

In the short term, planned cesarean section for persistent breech presentation is associated with a reduction in neonatal morbidity and mortality and a slight increase in maternal morbidity. It must be planned at 39 weeks with the intention of allowing the ideal physiological maturity of the fetus and the possibility of spontaneous version. Since spontaneous version can occur at any time, it is recommended to confirm the fetal position before the procedure, especially if the cesarean section is indicated only for breech presentation.^(11)^ A planned cesarean section policy may be inadequate or even unfeasible for places with limited resources. Furthermore, long-term maternal and child outcomes are similar in both modes of delivery, and cesarean sections potentially compromise and increase several risks in subsequent pregnancies (iteractivity, spectrum of placenta accreta, uterine rupture, maternal morbidity and mortality).^(13,14)^ It is estimated that 338 cesarean sections are necessary to avoid a single perinatal death.^(15)^ It is general consensus that pregnant women who choose to attempt labor and vaginal breech birth should be at low risk for complications and have assistance of professionals with experience in vaginal birth of anomalous presentations. The main focuses of the debate are related to the identification of these pregnancies and the comparison between the fetal risks of vaginal birth and the maternal risks of cesarean section. The progress of labor must be closely monitored and if inadequate, the threshold for indicating a cesarean section must be low. The adoption of strict protocols for planned vaginal breech birth correlates with low rates of adverse outcomes, and fetal and neonatal morbidity and mortality resemble that of planned cesarean section. The success rate of planned vaginal birth is approximately 70%, and adverse perinatal outcomes (brachial plexus injury, skull fracture, genital injury, intraventricular hemorrhage, seizures and death) have incidences of less than 7%. In addition, there is no evidence that an emergency cesarean section for a breech fetus is safer than a vaginal birth.^(16)^

## What are the main selection criteria for vaginal breech birth?

The adoption of the vaginal route in breech birth has had an important decrease, linked to the reduction in the teaching of skills and a large randomized clinical trial published at the beginning of this century. The definition of elective cesarean section at term as a safer and more appropriate procedure changed the teaching-care process and determined an important reduction in the acquisition of the necessary skills to assist the vaginal birth of breech fetuses.^(2,16,17)^ At the same time, corrections and new studies have better defined the selection criteria for the vaginal route of breech birth. Additionally, radiological evidence of enlargement of maternal pelvic diameters in non-lithotomy positions (all fours, squatting) associated with aortocaval decompression, optimization of uteroplacental blood flow and fetal oxygenation and experiences and reports on the safety and success of the vaginal route in these positions bring the interesting possibility of rescuing this mode of delivery, potentially as safe as never before reported.^(3,16)^ Most criteria for identifying pregnant women at lower risk for vaginal breech birth are based on expert opinions. There must be no obvious contraindications to the vaginal route of delivery, such as placenta previa. Pelvimetry (clinical or radiological) does not provide convincing evidence to select patients for vaginal breech birth. Previous cesarean section is a relative contraindication justified by the risks associated with labor attempts and vaginal breech birth after previous cesarean section. Cesarean sections with recurring indications must have the risks and benefits of an attempt at labor individually assessed.^(12)^ Prematurity imposes the risk of dystocia of the after-coming head motivated by the greater proportion of head circumference in relation to abdominal circumference (HC/AC). However, the gestational age at which this risk becomes significantly higher is not well defined. Although a gestational age above 36 weeks is safer, observational data on breech births between 32 and 36 weeks appear to favor the vaginal route due to reduced admissions to neonatal intensive care units, respiratory distress syndrome and use of antibiotics compared to cesarean sections.^(18)^ Even though there is recommendation against the induction of breech births, the maternal and perinatal morbidity and mortality of induced vaginal breech births appear to be similar to those of spontaneous vaginal and scheduled c-section births.^(12,16,18–20)^ A planned induction between 37 and 38 weeks performed by experienced professionals has a high success rate (close to 70%) and satisfactory neonatal results, which reinforces both the safety of the induction and the advantage of care provided by a specialized team working in places with resources for the safe performance of an emergency cesarean section (surgical facilities, team with anesthesia, obstetrics and pediatrics).^(21)^

Performing an ultrasound close to delivery is the criterion that should provide the greatest amount of information. Frank breech presentation or complete breech, those eligible for vaginal delivery, must be confirmed. There are no high-quality data to determine ideal weight limits and related risks for the vaginal route. The most frequently recommended weight range is 2,000 to 4,000 g. In intrauterine growth restriction, the risk of fetal acidosis due to chronic placental insufficiency may be aggravated by inevitable compression of the umbilical cord during the expulsion period, which is usually longer in breech presentations.^(22)^ Fetal anomalies that may cause dystocia (examples: macrocephaly and sacrococcygeal teratoma) must be excluded. Hyperextension of the fetal neck/head, defined as an angle of extension of the cervical spine ≥ 90° should not be present either, as it increases the risk of neurological injuries.^(23)^ Nulliparous women are considered high risk for vaginal breech birth by a significant portion of professionals under the justification of not yet having their pelvises tested. Despite data limitations, no differences were observed in neonatal outcomes between multiparous and nulliparous patients after planned vaginal breech birth.^(24)^ Therefore, pregnant women with fetuses in frank breech presentation or complete breech, weighing between 2,000 and 4,000 g, in the absence of intrauterine restriction of fetal growth, anomalies susceptible to dystocia and hyperextension of the cephalic pole, in spontaneous labor or under planned induction at term, with maternal desire and consent and an experienced obstetrician available, seem to be the most eligible for vaginal birth. Non-lithotomy positions should be encouraged, especially during the expulsion period. Pregnant women between 32 and 36 weeks, nulliparous women and those with previous cesarean sections can have a labor attempt individually assessed. Everyone must receive clear, objective and complete information. Ineligible patients who desire a vaginal birth have this right, hence should not be excluded from the possibility of an attempt and should receive the best possible assistance from the care team.^(12,16)^

## What are the main dystocias in vaginal breech birth care?

In the birth mechanism in breech presentation, most fetuses are positioned with their back anteriorly or rotate anteriorly during the descent. This is a more physiological mechanism, facilitates the release of the fetal trunk, which occurs with the fetal back facing the mother's abdomen, and provides adequate positioning of the occipital region of the fetus under the lower edge of the maternal pubic symphysis. However, in posterior position varieties (sacro-posterior), in which internal rotation occurs towards the posterior maternal pelvis, the fetal abdomen is positioned facing the maternal abdomen. This mechanism makes it difficult to release the fetal trunk (biacromial diameter) and leads to the fetal chin getting stuck in the maternal pubic symphysis, resulting in severe dystocia of the after-coming head. In these situations, timely obstetric intervention is necessary with the aim to prevent the rotation of the fetal back towards the posterior maternal pelvis, as well as difficulties in the release of the fetal biacromial and biparietal diameters.^(12,22)^ Deflection of one or both fetal arms adds undesirable volumes to the diameters of the cephalic pole, also creating difficulties in releasing the after-coming head. It is usually the result of untimely and incorrect traction performed in the absence of uterine contractions, causing fetal reflexes that result in elevation and stretching of the fetal arms and early deflection of the cephalic pole.^(12,22)^ In vaginal breech birth, cord prolapse umbilical cord is the most frequent complication associated with premature release of the lower limbs and amniorrhexis, occurring early before fetal delivery. When associated with difficulties in the delivery of the fetal trunk and/or head, the worsening of the already established hypoxic situation is significantly threatening.^(12,22)^

## What should be the conduct of care in vaginal breech births?

An obstetric clinical examination, accompanied or not by ultrasound, must be performed upon admission of the parturient woman with the aim to confirm the type of breech presentation and fetal attitude.^(1,12)^ With the objectives of both preventing umbilical cord prolapse and retention of the after-coming head, and favoring cervical effacement and dilation, the amniotic membranes must be kept intact, especially if it is a premature fetus birth. Ideally, amniorrhexis should occur only after fetal release has begun. The frequency of cord prolapse will be lower in the frank breech presentation. A vaginal examination should always be performed after spontaneous amniorrhexis.^(25)^ Electronic fetal monitoring must be continuous given the greater risk of umbilical cord compression. If available, an electrode can be adapted to the fetal buttock after amniorrhexis, when external monitoring is difficult.^(26)^ Neuroaxial analgesia is useful for pain relief and, when necessary, facilitates the performance of maneuvers and application of the forceps. When properly performed, it is effective and presents minimal motor blockage, preserving the ability to push and favoring maternal participation in fetal release. In its absence, a pudendal nerve block can be performed if operative vaginal delivery or episiotomy are necessary. Intravenous (IV) oxytocin can be used, especially in the prolonged latent phase and in transient post-anesthetic hypocontractility. As the prolongation of the active phase may indicate fetopelvic disproportion, its administration must be individualized in this situation.^(22)^ Buttock in De Lee plane 0 at 6 cm of dilation or occupying the pelvic floor at full dilation indicates adequate evolution. Delaying pushing until 90 minutes into the expulsion period appears to be acceptable in breech births.^(22)^ However, stopping descent after 60 minutes of pushing is indicative of a cesarean section, which will favor perinatal results.^(16)^ Non-lithotomic maternal positions (all fours, squatting) seem to be favorable, reducing interventions, expulsion time and neonatal injuries, although studies with a higher degree of scientific evidence are lacking.^(3,27)^ There is no scientific evidence guiding the practice of episiotomy in vaginal breech birth. Manipulation of the fetus can promote extension of the cephalic pole, making delivery difficult. No traction on the trunk, limbs or head should be applied, avoiding cervical and arm extension, the occurrence of nuchal arms and difficulties to release the upper limbs and cephalic pole. Therefore, there is a consensus that maneuvers should be avoided when assisting vaginal breech births, at least until the spontaneous release of the lower limbs and fetal abdomen. Specific maneuvers to rotate the trunk and extract the arms and head may be necessary, especially in the maternal lithotomy position.^(3,22,28)^ As neonatal morbidity is more common, pediatricians with experience in neonatology must be present at birth. Fetuses in abnormal presentations are more associated with anomalies and injuries during birth. The risk of hip dysplasia is also higher among them. Therefore, a complete neonatal examination is also necessary.^(29)^

## What should assistance be like for vaginal breech births in the lithotomy position?

**Figure 2:**
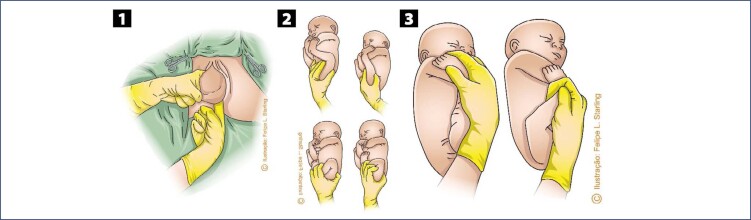
Maneuvers to help release the pelvis and lower limbs of the fetus in vaginal breech birth

Source: Illustrations by Felipe Lage Starling (authorized).

The difficulties in the sequential release of the bitrochanteric, biacromial and biparietal diameters are progressive. In the maternal lithotomy position, release will be slower than in vertical positions. During the descent and internal rotation of the pelvis, most fetuses will position their back upwards, facing superiorly towards the maternal abdomen. Therefore, the most proximal portions of the umbilical cord are positioned inferiorly to the fetal body, being compressed by it towards the maternal spine.^(12,22)^ Release of the fetal pelvic girdle usually does not cause problems. This process is slower in frank breech presentations, as the lower limbs, extended over the trunk, hinder the lateral inflection that promotes the regular exit of the bitrochanteric diameter. This difficulty can be resolved by means of bidigital inferior traction performed with the operator's fingers positioned in the fetal inguinal region (Figure 2).^(12,22,30)^ When there is an urgency to release the fetal pelvic girdle, foot traction corresponding to the anterior fetal hip ("good foot") is necessary. Therefore, it is necessary to pull the lower limb corresponding to the buttock that is closest to the anterior arch of the pelvis, facilitating the descent of the fetal pelvis under the maternal pubic arch, as performed in the internal version followed by foot extraction. In complete breech presentation, this procedure is simpler, as the fetal feet are more accessible. In frank breech presentations, the feet are towards the bottom of the uterus and may be more difficult to reach. This difficulty can be solved by using the Pinard maneuver. The operator's hand introduced into the maternal genitalia through the sacral void must ascend on the ventral surface of the fetus, looking for the popliteal fossa corresponding to the anterior hip. The operator's thumb wraps around the superior outer thigh, and the index and middle fingers are applied to the popliteal fossa. Flexion and abduction of the thigh are performed, facilitating the grip of the leg by the ring and little fingers, positioned anteriorly to the fetal ankle. Alternatively, the ventral hand can be deeply introduced into the uterine fundus, recognize the anterior foot, grasp it by the ankle with the index and middle fingers positioned in a hook and pull it inferiorly. These maneuvers allow the lowering of the anterior foot with subsequent extraction of the fetal hip (Figure 2).^(12,22,30)^

1. Bidigital inferior traction on the inguinal region. 2. Pinard maneuver. 3. Grip and inferior traction of the anterior fetal foot alternative to the Pinard maneuver.

Delivery of the fetal trunk can occur spontaneously, with progressive delivery of the abdomen, chest and upper limbs. For the most part, the birth mechanism occurs with the anterior rotation of the fetal back. During delivery of the fetal trunk, the biacromial diameter insinuates itself into the oblique diameter of the superior strait, descends and positions itself in the anteroposterior diameter of the inferior strait for subsequent expulsion of the anterior and posterior shoulders. With the spontaneous delivery of the fetal abdomen, gentle traction of the umbilical cord can be performed to partially remove it ("cord loop"), reducing funicular compression and providing better fetal oxygenation. During release, the trunk must be supported in a plane equal to or slightly lower than the horizontal plane of the birth canal. Meconium elimination is quite common.^(12,22)^

**Figure 3:**
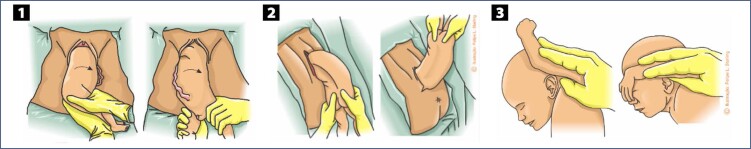
Maneuvers to help in delivery of the fetal trunk in vaginal breech birth

Source: Illustrations by Felipe Lage Starling (authorized).

In the absence of progression of the fetal trunk and arms, in the presence of fetal bradycardia or spontaneous and initial rotation of the fetal back towards the posterior side of the maternal pelvis, specific maneuvers are necessary. All of them must be performed without lower traction on the fetal body, preventing the fetus from abducting its shoulders and extending the arms upwards (nuchal arms), which potentially poses difficulties for the subsequent delivery of the head. The Rojas and Deventer-Miller maneuvers are used to release the abdomen and chest, and can be applied alternately. In the Rojas maneuver, the lower limbs of the fetus are grasped with the index, middle, ring and little fingers wrapped around each fetal thigh and the thumbs resting on the posterosuperior iliac spines (Figure 3). Rotational movements of 180° alternating in both directions are applied. Care must be taken to keep the fetal back facing the mother's abdomen at the end. In the Deventer-Miller maneuver, fetal grasp is identical. The fetus is positioned in the anteroposterior diameter and alternating movements of raising and lowering the trunk are applied, like a pendulum (Figure 3). The sequencing of these two maneuvers is also described as the Lovset maneuver. The Pajot maneuver can be used to release the fetal shoulders and arms, especially when they are raised. Each fetal shoulder must be grasped from behind, with the operator placing his or her hand opposite the shoulder to be extracted. The thumb rests on the armpit, the "arch" of the hand rests on the shoulder and the index and middle fingers are positioned parallel to the humerus. Arm traction is performed inferiorly and anteriorly to the fetal body. The elbow and forearm slide in front of the fetal face downwards to the chest, allowing the arm to be released (Figure 3).^(12,22,30)^

1. Rojas maneuver. 2. Deventer-Miller maneuver. 3. Pajot maneuver.

**Figure 4:**
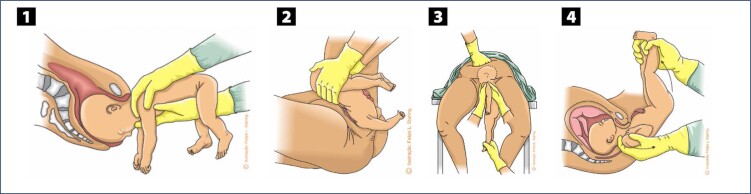
Maneuvers to help release the after-coming head

Source: Illustrations by Felipe Lage Starling (authorized).

In lithotomy position, delivery of the after-coming head with the aid of maneuvers is commonly necessary. In the Mauriceau-Smellie-Veit (or Mauriceau) maneuver, the fetal thorax and abdomen are positioned over the operator's inferior arm. The index and ring fingers of the lower hand are positioned on the malar eminences and the middle finger on the maxilla, aiming to flex the cephalic pole. Alternatively, the index and middle fingers can be positioned at the base of the fetal tongue. With the other arm positioned above the fetal back, the upper hand grasps the shoulders from behind with the index and ring fingers on each side of the fetal neck and the middle finger positioned on the occiput to exert counterpressure. Release is achieved through simultaneous elevation of the arms, maintaining flexion of the fetal cephalic pole until delivery is completed (Figure 4). While performing the maneuver, the fetal body must not be displaced more than 45° above the horizontal maternal plane, preventing hyperextension of the cervical spine and occlusion of the vertebral arteries.^(30,31)^

1. Mauriceau maneuver. 2. Bracht maneuver. 3. Champetier de Ribes maneuver. 4. Prague maneuver.

In the Bracht maneuver, the fetal body is grasped with support of the thumbs on the posterosuperior iliac spines, and the thighs and back are involved with the other fingers. The fetal trunk is elevated and projected towards the maternal abdomen, maintaining flexion of the thighs over the abdomen during execution of the maneuver (Figure 4).^(28,30)^

In the face of the difficulty in releasing the after-coming head, the insinuation of the cephalic pole can be obtained with the Liverpool maneuver. The fetal body is left in a pending position for 20 seconds, and attempts at delivery with other maneuvers are subsequently resumed.^(22)^

The Champetier de Ribes maneuver should be used to release the after-coming head from a supposedly flat pelvis, and is recommended after failure of the initial maneuvers. The parturient woman should be positioned with the buttocks close to the edge of the birthing bed (or stretcher) and the lower limbs outside the leg holders, hanging below the level of the pelvis and projected towards the floor (Crouzat-Walcher position) with the intention of expanding the superior strait. This position promotes counternutation of the sacrum, lifting the lumbosacral joint against gravity and increasing the anteroposterior diameter of the posterior pelvis. With the index and ring fingers of the hand positioned in front of the fetal thorax, the first operator grasps the fetal malar (or alternatively, the base of the fetal tongue) and flexes the fetal cephalic pole and rotates it towards the transverse diameter of the pelvis. Simultaneously, this first operator places the index and middle fingers of the other hand on the fetal back, grasping the fetal shoulders from behind. This operator will command the actions. The second operator grasps the fetus’ feet and the third places a hand on the uterine fundus. With the first operator keeping the fetal cephalic pole in flexion and the third exerting manual pressure on the uterine fundus, both continuously, the second operator lifts the fetal body by the feet, projecting it towards the maternal thorax, with the aim to release the posterior parietal part of the fetus. Sequentially, the second operator projects the fetal body in the opposite direction, that is, towards the floor and maternal lower limbs, aiming at the release of the anterior fetal parietal (Figure 4).^(30)^

The Wigand-Martin-Winckel maneuver is a variant of the Champetier de Ribes process and is recommended in the absence of assistants. The operator performs flexion and rotation of the cephalic pole towards the transverse diameter using only one hand (the ventral hand). The middle finger is positioned inside the fetus's mouth at the base of the tongue. The index and ring fingers are supported on the malar, on each side of the nasal pyramid. The thumb converges towards the jaw. The other hand, positioned externally, exerts abdominal pressure on the cephalic pole, assisting in the process of insinuation, descent and release of the parietals.^(30,32)^

Dystocia of the after-coming head of the fetus whose body is released with the ventral side facing the mother's abdomen is difficult to manage. The Prague maneuver is the most recommended in this situation. One of the operator's hands positioned inferiorly and supporting the fetal body from the back grasps the shoulders with the index and ring fingers positioned next to the neck and the middle finger exerting counterpressure on the occiput. The operator's other hand grasps the lower limbs of the fetus at ankle height. Sequentially, the hand applied to the ankles elevates the lower limbs, projecting them towards the maternal abdomen, while the hand applied to the shoulders performs axial traction, aiming at delivery of the fetal cephalic pole (Figure 4).^(30)^

**Figure 5:**
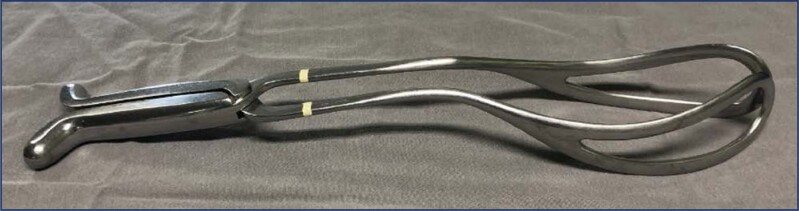
Piper's forceps

Source: Photographic record by the authors.

The failure of these specific maneuvers imposes the need for operative vaginal delivery to release the after-coming head. The use of a vacuum extractor is impossible, as there is no accessibility to the flexion point located between the fontanelles (bregma and lambda). Therefore, forceps are the instrument of choice for the operation, and the Piper's forceps is specific to the situation. It has long (44 cm long) and crossed blades, English articulation and handles without fingerings and fins. Its spoons are fenestrated and have very prominent cephalic and pelvic curvatures. A third curvature, the perineal, is present on the lower surface of the stems, close to the spoons. The perineal curvature is specific of this instrument and was designed to avoid the need for excessive elevation (>45°) of the fetal body above the instrument (Figure 5).^(33,34)^

**Figure 6:**
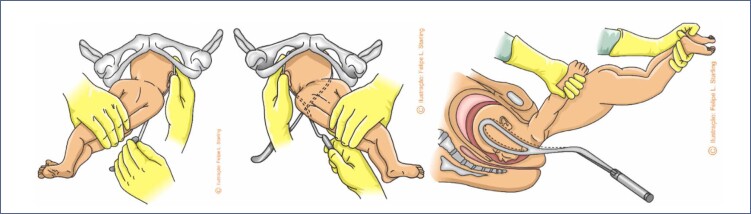
Application of Piper's forceps to the after-coming head

Source: Illustrations by Felipe Lage Starling (authorized).

Before introducing the forceps blades, the operator positions the fetal cephalic pole in occiput posterior (OP) by applying the index and middle fingers to the base of the fetal tongue. To apply the forceps to the after coming head in the anterior variety (fetal back facing the mother's abdomen), an assistant moves the fetal body laterally, grasping it by the wrists and ankles. The operator, kneeling, introduces the forceps blades underneath the fetal body in a horizontal position, applying them to the parietal bones. As in breech presentation the diameters of the skull that are accessible at the vulva are smaller than the parietal diameters that are more superior, the operator's hand that serves as a guide must go deep into the vagina to obtain the correct insertion and articulation of the blades. The left blade ("female" blade) is applied first and then, the right blade ("male" blade) in order to avoid uncrossing the forceps blades. After articulating the blades of the forceps, the criteria for good grip are checked, confirming the equidistance of the facial line in relation to the blades of the forceps and the impossibility of passing the fingers inside the fenestrae of the spoons. In the sitting position, the operator flexes the fetal head without traction, placing the suboccipital region of the fetus under the pubic arch. Delivery of the head is performed by accentuating the flexion with the instrument articulated. Simultaneously, the assistant elevates the fetal body and, optionally, another assistant performs manual pressure on the uterine fundus (Figure 6).^(33,34)^

In posterior varieties, that is, when the fetal back is inadvertently positioned in the direction of the maternal back, the forceps blades must be introduced above the fetal body, and the application is carried out in the occiput anterior (OA). Traction is exerted forward with the fetal jaw and neck resting on the upper edge of the pubic symphysis. The fetal trunk is then elevated towards the maternal abdomen. In the absence of Piper's forceps, the longer Simpson's forceps (35 cm) or Kielland's forceps are the instruments of choice to replace it.^(30,33)^

Persistent dystocia of the after-coming head is a potentially serious complication of breech birth and may require rescue procedures. The patient must be positioned in McRoberts by hyperflexing the lower limbs against the abdomen. Some uterine and cervical relaxant should be administered. Options include beta-adrenergic agonists (salbutamol 0.05 mg in slow IV infusion; terbutaline 0.25 mg subcutaneously), nitroglycerin (0.05 to 0.2 mg IV) or even induction of general anesthesia. Other procedures that pose greater maternal-fetal risk include Dührssen incisions, the Zavanelli maneuver and symphysiotomy.^(22,30)^

Especially in premature babies, the retention of the after-coming head in the cervix can be resolved through Dührssen incisions. One or two fingers are introduced between the cephalic pole and the cervix. Incisions are made with scissors and must extend the entire remaining length of the cervix and not extend into the lower uterine segment or broad ligament. Between one and three incisions are made. The first incision should be made at the six o'clock position. If necessary, incisions at the two and ten o'clock positions should also be made. Incisions at the three and nine o'clock positions are not recommended to prevent injuries to the cervicouterine arteries.^(22)^

The Zavanelli maneuver followed by cesarean section can also be attempted. Its execution is more difficult than in shoulder dystocia, as the entire fetal trunk and limbs must be pushed back in the uterus.^(30,35)^

Symphysiotomy should be an exceptional maneuver, as it can be associated with various morbidities (vaginal and urinary tract lacerations, urinary incontinence, vesicovaginal fistula) and long-term pelvic instability. It should only be performed when other procedures fail and in places without operating rooms. The technique is performed under local anesthesia with the parturient in lithotomy and lower limbs abducted. After bladder catheterization, the anesthetic must be infiltrated into the skin and subcutaneous tissue overlying the pubic cartilage. The urethra is deviated laterally with one hand and a 1-3 cm incision is made with a scalpel blade. The incision must be sufficient to divide the pubic symphysis and release the fetal head. It is not necessary to incise the full thickness of the cartilage, as a modest separation of the pubic symphysis, which will be permanent, is sufficient to release the cephalic pole. After the procedure, absolute rest is recommended for two days, followed by progressive mobilization. Abduction of the lower limbs should be avoided for seven to ten days.^(22,36)^

## What should assistance be like for vaginal breech births in non-lithotomy positions?

**Figure 7:**
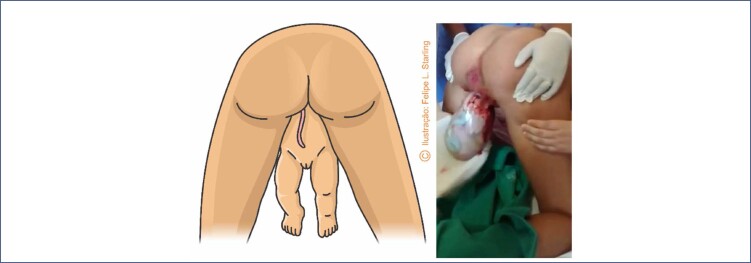
View of vaginal breech birth in the all fours position

Source: Illustration by Felipe Lage Starling (authorized); photographic record by the authors.

**Figure 8:**
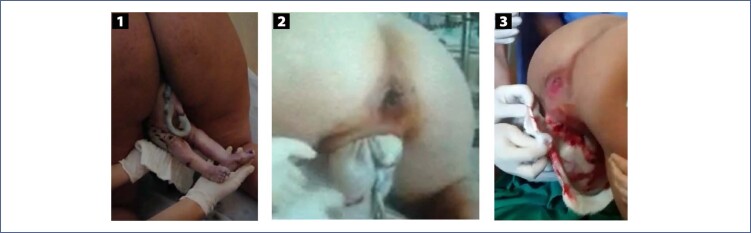
Aspects to be observed during vaginal breech birth in the all fours position

Source: Photographic record kindly provided by Dr. Caroline Reis Gonçalves; photographic records by the authors.

There are no randomized clinical trials comparing maternal and perinatal outcomes of assisted vaginal breech birth in vertical positions (all fours, squatting, sitting) with those in horizontal positions (supine, lithotomy, lateral decubitus). Current knowledge is based on ergonomic studies and the experience and opinions of experts.^(3,37)^ Compared to lithotomy, breech birth in other maternal positions is associated with a reduction in the dilation period, rate of cesarean sections, need for maneuvers for fetal extraction and rate of neonatal injuries. Among women in labor in the all fours position (Gaskin's position), the delivery of more than half of breech fetuses occurs without the need for any maneuver, and this position allows good observation of the progressive expulsion of fetal segments. The all fours position in breech birth has introduced a new understanding of the cardinal movements of the birth mechanism. It also reduced the use of maneuvers to assist fetal release, usually necessary in the supine position and in cesarean sections, and provided the description of new maneuvers specific to this maternal position.^(3)^ Assistance for pelvic-vaginal birth in the all fours position is eminently observational. The assistant must know the delivery mechanism and the signs indicating physiological progression, avoiding the unnecessary use of maneuvers. Under no circumstances should the fetus be pulled during its release. The few maneuvers that may be necessary are to perform rotations and improve the positioning of fetal diameters without exerting vertical traction. After release of the bitrochanteric diameter, the fetus must rotate its back anteriorly (towards the maternal abdomen) and the "birth vision" is integrated by the maternal back and fetal abdomen (Figure 7). The fetal abdomen, positioned superiorly, reduces funicular compression. The tone of the fetal lower limbs, already released, and the engorgement of the umbilical cord must be observed (Figure 8). Observation of an engorged umbilical cord infers good circulation and, therefore, adequate fetal oxygenation. A pale, bloodless cord is a warning of fetal hypoxia and indicative of the need for maneuvers to promote the complete delivery. As the abdomen is released, the presence of the elbows and/or fold in the fetal chest should be observed, indicating the maintenance of the fetal arms flexed in front of the chest and the absence of shoulder dystocia (Figure 8). A small physiological and spontaneous rotation may occur during the release of the arms. During this process, it is very common for the woman in labor to flex her knees, bringing the lower limbs of the fetus, already released, closer to the lower support surface where the birth is being assisted. Contact of the fetal lower limbs with a flat surface usually triggers a flexion reflex of the abdomen ("tummie crunch"), which should not be restrained. Flexion of the fetal abdomen favors flexion of the cephalic pole, which is still located in the lower strait of the maternal pelvis. After delivery of the biacromial diameter and upper limbs, maternal anal dilation is indicative of the descent of the flexed cephalic pole and its imminent release (Figure 8). The absence of maternal anal protrusion indicates the need for a maneuver to correct the flexion of the fetal cephalic pole ("Frank's nudge").^(3)^

1. Fetal lower limb tone and engorged umbilical cord. 2. Presence of elbows and folds of the fetal chest. 3. Maternal anal dilation.

**Figure 9:**
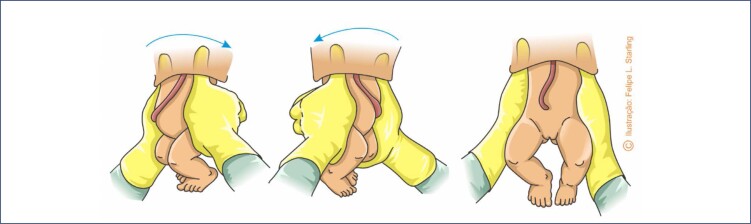
Maneuver of "180°-90° rotation" to release the shoulders in vaginal breech birth in the all fours position

Source: Illustration by Felipe Lage Starling (authorized).

Vaginal breech birth assisted in the all fours position reduced the number of maneuvers to practically two: one to help release the shoulders and another to release the cephalic pole. Both should only be performed if there is no expulsive progression or if fetal hypoxia is suspected. In the event of "nuchal arms" or shoulder dystocia, the fetus positions the biacromial diameter in the oblique or anteroposterior diameters of the maternal pelvis, interrupts its descent and release, and remains with the trunk positioned laterally. Incorrect rotation of the fetal back may also occur, in which it is positioned in the assistant's view. The "180°-90° rotation" maneuver is indicated to correct these dystocias. The fetal thorax is grasped by applying the thumbs to the ventral portion of the shoulders (over the fetal clavicles) and the other fingers to the fetal scapulae. Then, the fetal body is rotated 180° away from the fetal abdomen. Sequentially, the fetus is rotated back to the previous position, but only by 90°. Therefore, at the end of the rotations, the fetus must occupy the transverse diameter of the pelvis, with its abdomen facing the operator (Figure 9). Care must be taken not to perform this maneuver by grasping the fetal abdomen alone, which is not very effective and potentially harmful. Since the rotational maneuver is effective, the cephalic pole release maneuver should be carried out immediately afterwards. If the "180°-90° rotation" maneuver fails, the Pajot maneuver must be attempted for the complete delivery of the biacromial diameter.^(3)^

**Figure 10:**
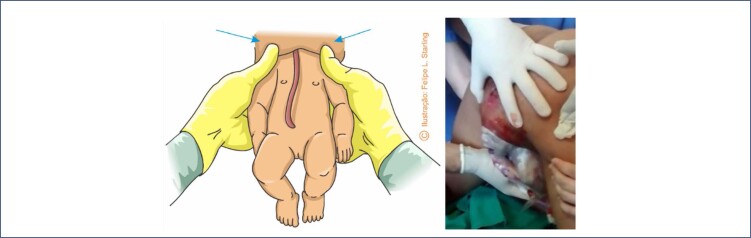
"Frank's nudge" maneuver to help release the cephalic pole in vaginal breech birth in the all fours position

Source: Illustration by Felipe Lage Starling (authorized); photographic record by the authors.

When the "180°-90° rotation" maneuver is performed or there are signs of the after-coming head (non-dilated maternal anus), delivery of the cephalic pole can be assisted with the "Frank's nudge" maneuver. With the hands arranged in the same way as the "180°-90° rotation" maneuver, the fetal trunk is "pushed" backwards towards the mother's belly. This maneuver pushes the occiput of the fetus against the mother's pubis, providing support for the flexion of the cephalic pole, bringing the chin closer to the sternum and finalizing the expulsion. Alternatively, one of the hands flat presses the fetal thorax backwards (towards the mother's belly), while the other rests on the perineum (Figure 10).^(3)^

**Figure 11:**
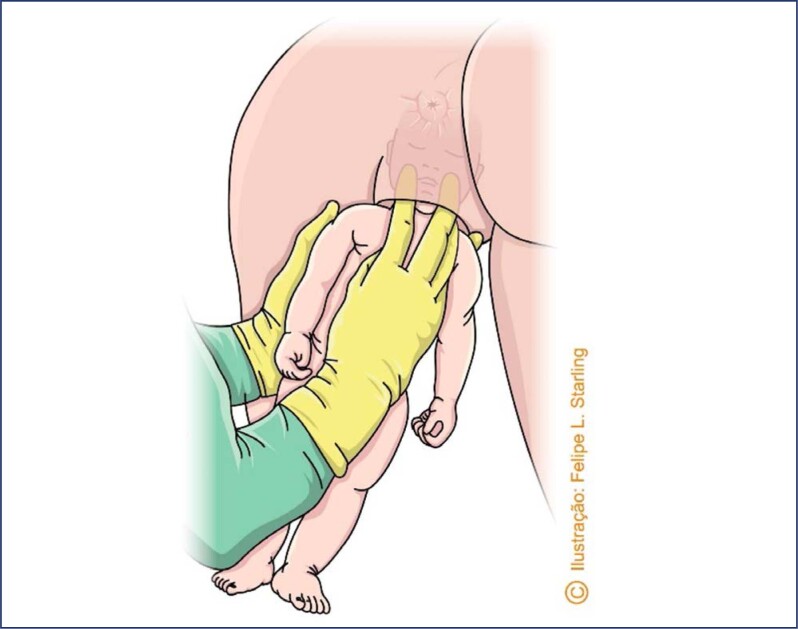
Mauriceau-Cronk maneuver to help release the cephalic pole in vaginal breech birth in the all fours position

Source: Illustration by Felipe Lage Starling (authorized).

In the failure of the "Frank's nudge" maneuver to promote flexion of the fetal cephalic pole, the Mauriceau-Cronk maneuver may resolve the deflection. The operator places the index and middle fingers of the dominant hand on the fetal cheekbones. The index, middle and ring fingers of the other hand are positioned on the fetal occiput, forcing the head to flex, while it is simultaneously pushed against the maternal pubis (Figure 11). If the cephalic pole is not released after this attempt, the parturient must be positioned in lithotomy for other maneuvers or application of Piper's forceps.^(38)^

## What should a cesarean section be like in breech presentation?

In order to provide time for spontaneous cephalic version and reduce the likelihood of neonatal respiratory problems, pregnant women who are not eligible for vaginal breech birth should have a cesarean section scheduled between 39+0 and 41+0 weeks or undergo unscheduled surgery at the onset of spontaneous labor. Confirmation of fetal position, preferably by ultrasound, should be carried out immediately before surgery. In situations where premature birth is indicated for medical reasons, a cesarean section must be scheduled. In pregnant women in spontaneous premature labor, a cesarean section should be performed if signs of inevitability of labor are present.^(12,22)^ The abdominal incision and hysterotomy must be wide, avoiding difficult and traumatic fetal extractions. Low transverse, arciform, superior cavus segmental hysterotomy (Fuchs-Marshall) is suitable for the extraction of term and borderline fetuses. For premature babies, especially extremely premature babies, a transverse hysterotomy in a narrow segment can lead to difficulty in fetal extraction. In these situations, longitudinal segmental hysterotomy (Krönig) or corporal hysterotomy defined through clinical judgment facilitate fetal extraction and can be optimized with pharmacological uterine relaxation (beta-adrenergic agonists, nitroglycerin, halothane). The maneuvers are the same as those used to assist vaginal breech births in the lithotomy position, and fetal extraction must be as delicate and atraumatic as possible. Appropriately sized forceps should also be available. Hyperextension and/or vigorous traction on the cervical spine must be avoided during delivery of the cephalic pole.^(39)^

## What should assistance be like in special situations of breech birth?

**Figure 12:**
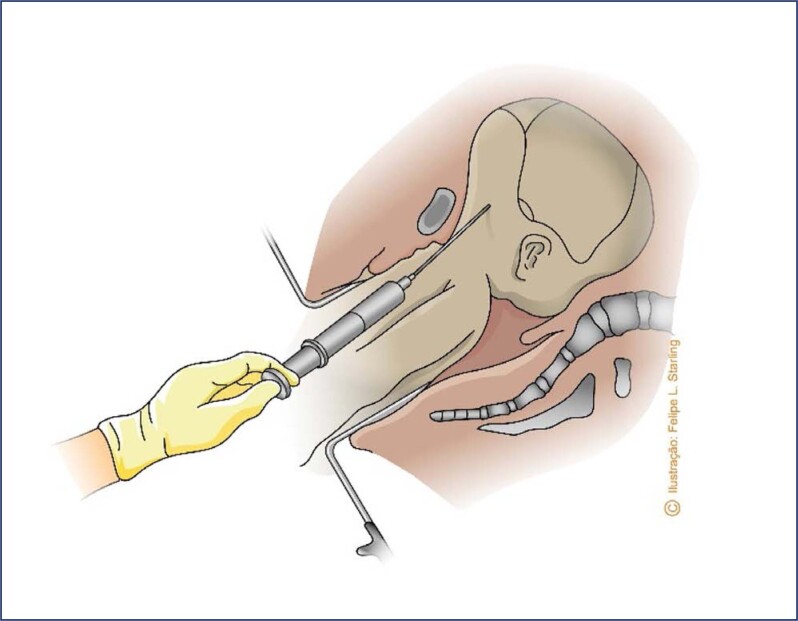
Cephalocentesis in the vaginal route of breech delivery of a non-viable fetus with hydrocephalus and macrocephaly

Source: Illustration by Felipe Lage Starling (authorized).

Breech presentation is commonly associated with fetal anomalies, particularly macrocephalic fetuses. Therefore, the diagnosis and individualized study of fetal anomalies that can cause dystocia (hydrocephalus with macrocephaly, cystic hygromas, other large tumors) and of fetal viability are necessary for defining the route of delivery. Viable breech fetuses with significant hydrocephalus and macrocephaly should be extracted abdominally, avoiding a severe entrapment of the after-coming head. The cesarean section must have enlarged abdominal and uterine incisions; longitudinal segmental hysterotomy (Krönig) is preferable, with the possibility of extension to the upper segment or uterine body. Fetuses in this situation, although unviable, should preferably be selected for vaginal delivery, as, in addition to perinatal asphyxia not influencing the prognosis of a lethal congenital anomaly, this method of delivery is safer for the mother. In case of fetal head entrapment, a cephalocentesis may be necessary to decompress and collapse the skullcap. The procedure can be performed either abdominally or vaginally, using a large-gauge spinal needle (no. 16 or 18). Preferably, the puncture should occur when the head is already fixed in the pelvis, after expulsion of the fetal trunk and limbs, a situation in which the fetus is usually no longer alive. The fetus must be vigorously pulled, exposing the base of the skull. The needle is penetrated into the ventricular cavity through the occiput (in the sutures) or through the foramen magnum (Figure 12). A Piper's forceps may be necessary to complete the extraction of the cephalic pole. In fetuses with associated spina bifida, an alternative is intraventricular catheterization with a long metallic or urethral probe. The probe is introduced through the spina bifida until it reaches the cranial cavity.^(38)^

In breech births progressing to the vaginal route and assistance without qualified staff and/or adequate facilities, guidance against voluntary pushing, pharmacological uterine relaxation (for example: terbutaline 0.25 mg subcutaneously) and assessment of the possibility of transfer of care are recommended. If the birth progresses with imminent or expulsion of the buttocks or lower limbs, in addition to guidance against the traction of the fetus, verticalization of the parturient in the all fours position or squatting position is recommended. In most cases, fetal expulsion will be spontaneous, as previously detailed.^(38)^

## Final considerations

At the same time, assistance during pregnancy and delivery of breech fetuses has evolved with drastic changes in its conduct. The disuse of ECV and the increasingly frequent use of the abdominal route of birth inherent to the detriment of acquiring the obstetric surgical skills necessary to assist these vaginal births are notable and concomitant with emergency situations. Admissions of parturient women in more advanced stages of the expulsion period have not ceased to exist, and often occur in the face of unprepared teams nowadays. At the same time, evidence of the benefits of properly selecting parturient women for the vaginal route of delivery, as well as the adoption of upright positions during care offer the specialty the possibility of rescuing this route of delivery and pose a great challenge for those who still have these skills and are dedicated to teaching obstetric surgery.

## National Specialized Commission on Obstetric Emergencies of the Brazilian Federation of Gynecology and Obstetrics Associations (Febrasgo)

**President:**

Álvaro Luiz Lage Alves

**Members:**

Gabriel Costa Osanan
Samira El Maerrawi Tebecherane Haddad
Adriana Amorim Francisco
Alexandre Massao Nozaki
Brena Carvalho Pinto de Melo
Breno José Acauan Filho
Carla Betina Andreucci Polido
Eduardo Cordioli
Frederico José Amedée Peret
Gilberto Nagahama
Laíses Braga Vieira
Lucas Barbosa da Silva
Marcelo Guimarães Rodrigues
Rodrigo Dias Nunes
Roxana Knobel

## References

[1] ACOG Committee Opinion No. 745: mode of term singleton breech delivery (2018). Obstet Gynecol.

[2] Hehir MP (2015). Trends in vaginal breech delivery. J Epidemiol Community Health.

[3] Louwen F, Daviss BA, Johnson KC, Reitter A (2017). Does breech delivery in an upright position instead of on the back improve outcomes and avoid cesareans?. Int J Gynaecol Obstet.

[4] Casteels M, Podevyn K, Vanoverschelde H, Louwen F (2021). Implementation of a breech program in a Belgian obstetric team. Int J Gynaecol Obstet.

[5] Jennewein L, Allert R, Möllmann CJ, Paul B, Kielland-Kaisen U, Raimann FJ (2019). The influence of the fetal leg position on the outcome in vaginally intended deliveries out of breech presentation at term – A FRABAT prospective cohort study. PLoS One.

[6] Hickok DE, Gordon DC, Milberg JA, Williams MA, Daling JR (1992). The frequency of breech presentation by gestacional age at birth: a large population-based study. Am J Obstet Gynecol.

[7] Toijonen AE, Heinonen ST, Gissler MV, Macharey G (2020). A comparison of risk factors for breech presentation in preterm and term labor: a nationwide, population-based case-control study. Arch Gynecol Obstet.

[8] Ford JB, Roberts CL, Nassar N, Giles W, Morris JM (2010). Recurrence of breech presentation in consecutive pregnancies. BJOG.

[9] Westgren M, Edvall H, Nordström L, Svalenius E, Ranstam J (1985). Spontaneous cephalic version of breech presentation in the last trimester. Br J Obstet Gynaecol.

[10] Hofmeyr GJ, Kulier R, West HM (2015). External cephalic version for breech presentation at term. Cochrane Database Syst Rev.

[11] Hofmeyr GJ, Hannah M, Lawrie TA (2015). Planned caesarean section for term breech delivery. Cochrane Database Syst Rev.

[12] Sentilhes L, Schmitz T, Azria E, Gallot D, Ducarme G, Korb D (2020). Breech presentation: clinical practice guidelines from the French College of Gynaecologists and Obstetricians (CNGOF). Eur J Obstet Gynecol Reprod Biol.

[13] Whyte H, Hannah ME, Saigal S, Hannah WJ, Hewson S, Amankwah K (2004). Outcomes of children at 2 years after planned cesarean birth versus planned vaginal birth for breech presentation at term: the International Randomized Term Breech Trial. Am J Obstet Gynecol.

[14] Nordborg JW, Svanberg T, Strandell A, Carlsson Y (2022). Term breech presentation-intended cesarean section versus intended vaginal delivery-a systematic review and meta-analysis. Acta Obstet Gynecol Scand.

[15] Vlemmix F, Bergenhenegouwen L, Schaaf JM, Ensing S, Rosman AN, Ravelli AC (2014). Term breech deliveries in the Netherlands: did the increased cesarean rate affect neonatal outcome? A population-based cohort study. Acta Obstet Gynecol Scand.

[16] Goffinet F, Carayol M, Foidart JM, Alexander S, Uzan S, Subtil D (2006). Is planned vaginal delivery for breech presentation at term still an option? Results of an observational prospective survey in France and Belgium. Am J Obstet Gynecol.

[17] Hannah ME, Hannah WJ, Hewson SA, Hodnett ED, Saigal S, Willan AR (2000). Planned caesarean section versus planned vaginal birth for breech presentation at term: a randomised multicentre trial. Term Breech Trial Collaborative Group. Lancet.

[18] Toijonen A, Heinonen S, Gissler M, Macharey G (2022). Neonatal outcome in vaginal breech labor at 32 + 0-36 + 0 weeks of gestation: a nationwide, population-based record linkage study. BMC Pregnancy Childbirth.

[19] Gaillard T, Girault A, Alexander S, Goffinet F, Le Ray C (2019). Is induction of labor a reasonable option for breech presentation?. Acta Obstet Gynecol Scand.

[20] Welle-Strand JA, Tappert C, Eggebø TM (2021). Induction of labor in breech presentations - a retrospective cohort study. Acta Obstet Gynecol Scand.

[21] Owada M, Suzuki S (2021). Outcomes of "one-day trial of vaginal breech delivery of singleton pregnancy" at 37-38 weeks’ gestation at a Japanese perinatal center. J Matern Fetal Neonatal Med.

[22] Kotaska A, Menticoglou S, Gagnon R, Maternal Fetal Medicine Committee (2009). Vaginal delivery of breech presentation. J Obstet Gynaecol Can.

[23] Westgren M, Grundsell H, Ingemarsson I, Mühlow A, Svenningsen NW (1981). Hyperextension of the fetal head in breech presentation. A study with long-term follow-up. Br J Obstet Gynaecol.

[24] Kielland-Kaisen U, Paul B, Jennewein L, Klemt A, Möllmann CJ, Bock N (2020). Maternal and neonatal outcome after vaginal breech delivery of nulliparous versus multiparous women of singletons at term-a prospective evaluation of the Frankfurt breech at term cohort (FRABAT). Eur J Obstet Gynecol Reprod Biol.

[25] Girault A, Carteau M, Kefelian F, Menard S, Goffinet F, Le Ray C (2022). Benefits of the «encaul» technique for extremely preterm breech vaginal delivery. J Gynecol Obstet Hum Reprod.

[26] Jettestad MC, Schiøtz HA, Yli BM, Kessler J (2019). Fetal monitoring in term breech labor - a review. Eur J Obstet Gynecol Reprod Biol.

[27] Bogner G, Strobl M, Schausberger C, Fischer T, Reisenberger K, Jacobs VR (2015). Breech delivery in the all fours position: a prospective observational comparative study with classic assistance. J Perinat Med.

[28] Jotkowitz MW, Picton FC (1970). An appraisal of an anatomically and physiologically correct method of breech delivery: the Bracht manoeuvre. Aust N Z J Obstet Gynaecol.

[29] de Hundt M, Vlemmix F, Bais JM, Hutton EK, de Groot CJ, Mol BW (2012). Risk factors for developmental dysplasia of the hip: a meta-analysis. Eur J Obstet Gynecol Reprod Biol.

[30] Rezende J (1998). Versão. Extração podal.

[31] Grosfeld O, Kretowicz J, Brokowski J (1980). The temporomandibular joint in children after breech delivery. J Oral Rehabil.

[32] Wigand JH, Naegele FK (1820). Die Geburt des Menschen, in physiologisch-diätetischer und pathologisch-therapeutischer Beziehung.

[33] Benzecry R (2006). Fórcipe passo a passo.

[34] Piper EB, Bachman C (1972). The prevention of fetal injuries in breech delivery. Journal of the American Medical Association, vol. 92, pp. 217-221, 1929. Am J Obstet Gynecol.

[35] Sandberg EC (1999). The Zavanelli maneuver: 12 years of recorded experience. Obstet Gynecol.

[36] Wilson A, Truchanowicz EG, Elmoghazy D, MacArthur C, Coomarasamy A (2016). Symphysiotomy for obstructed labour: a systematic review and meta-analysis. BJOG.

[37] Hofmeyr GJ (2023). Delivery of the singleton fetus in breech presentation [Internet].

[38] Cronk M (1998). Keep your hands off the breech. AIMS J Aut.

[39] Westgren LM, Songster G, Paul RH (1985). Preterm breech delivery: another retrospective study. Obstet Gynecol.

